# Extensive HPV Genotyping Reveals High Association between Multiple Infections and Cervical Lesions in Chinese Women

**DOI:** 10.1155/2022/8130373

**Published:** 2022-06-10

**Authors:** Fangfang Zhong, Ting Yu, Xiaoxi Ma, Shunni Wang, Qing Cong, Xiang Tao

**Affiliations:** ^1^Department of Pathology, Obstetrics and Gynecology Hospital of Fudan University, Shanghai 200090, China; ^2^Center for Diagnosis and Treatment of Cervical Diseases, Obstetrics and Gynecology Hospital of Fudan University, Shanghai 200090, China

## Abstract

**Objective:**

The relationship between human papillomavirus (HPV) and cervical lesions has been extensively elucidated, but infection with multiple genotypes is less investigated due to methodology limitations. In the current study, with a method of genotyping 21 HPVs in a routine cervical screening population, we aimed to investigate the prevalence and diversity of HPV infections in Chinese women and further evaluate the impact of multiple infections of HPV on cervical lesion progression.

**Methods:**

Totally, 73,596 patients who underwent 21-genotype HPV testing from January 2018 to April 2019 were retrieved from the database of the Department of Pathology, Obstetrics and Gynecology Hospital of Fudan University. HPV testing was performed by real-time PCR assay, including 13 high-risk HPVs (hrHPV), 5 potential hrHPVs, and 3 low-risk HPVs.

**Results:**

Of the 17,079 (infection rate, 23.2%) hrHPV- or potential hrHPV- (hr/phrHPV-) positive cases, 26.3% had multiple infections. Women younger than 25 and older than 65 were more prone to multiple infections. Of the hr/phrHPV-positive cases involving cervical intraepithelial neoplasia (CIN) 2 or worse (CIN2+), HPV73, 53, and 66 (=59) were the top three genotypes most likely to be included in multiple infections, while HPV16, 18, and 58 were the 3 least. Patients with single infection of HPV16 had higher incidences of CIN2+ than those with multiple-infection pattern (*P* < 0.001), indicating that mixing with other genotypes alleviated pathogenicity. The infection of HPV52, 53, 56, 51, 39, 66, 59, 68, and 35 showed an opposite pattern, indicating that they were less likely to be pathogens individually. All other types showed no significant differences, indicating the capability of pathogenesis independently. HPV26 showed a higher OR for CIN2+ than most traditional hrHPV genotypes. The vial load and the percentage of HPV16 showed positive correlation with the severity of cervical lesions.

**Conclusion:**

Extensive genotyping identified 3 most frequent genotypes, HPV16, 52, and 58, in CIN2+ of Chinese population. HPV16 mixing with other genotypes alleviated its pathogenicity. The vial load and the percentage of HPV16 were positively correlated with the severity of cervical lesions. HPV26 may be considered as a hrHPV, which needs to be evaluated and confirmed by more cases.

## 1. Introduction

Cervical cancer has the highest incidence of the three major malignant tumors in female reproductive system worldwide. There are 604,127 new cases of cervical cancer worldwide in 2020, of which 109,741 are from China [1]. In addition to the fact that China is a populous country, the most important reasons for the large number of new cases of cervical cancer in China include the neglection of cervical cancer screening in the past few decades together with the late start and insufficient popularization of HPV vaccination. Human papillomavirus (HPV) detection is included in various screening strategies for cervical cancer and precancerous lesions. It provides a more sensitive method of detection for high-grade lesions than cytology [2]. And it is recommended as the preferred screening method for cervical cancer screening by the latest WHO guidelines [3]. HPV infections were significantly region-specific, and the prevalence of variable hrHPV genotypes infection varies geographically [4, 5]. A systematic review on the epidemiological studies published from January 2000 to June 2018 on high-risk HPVs in mainland Chinese women has shown that the main high-risk HPV subtypes in women of mainland China were 16, 52, 58, 53, and 18, all of which were significantly region- and age-specific [6]. They found that the overall infection rate of hrHPV was highest (23.8%) in the north of China and lowest (12.2%) in the northwest of China, and the top 5 subtypes of which were 16, 58, 52, 18, and 33 and 16, 58, 53, 52, and 51, respectively [6]. However, in 2021, a study on the nationwide prevalence and genotype distribution of hrHPV showed that the overall hrHPV infection rate was 19.1%, and the top 6 types were 52, 16, 53, 58, 51, and 68. Overall, hrHPV-positive results varied regionally from 15.3% to 24.4% [4]. It suggested that the overall epidemic characteristics of HPV infection in Chinese women are similar in recent years, but still region-specific.

The relations between the virus and cervical lesions have been extensively elucidated, but multiple-genotype infections are less investigated due to methodology limitations. The results from various population studies have shown that multiple-HPV-genotype infections are frequently encountered, and cervical lesions are not always caused by a single HPV genotype [7–10]. Different studies have produced different results regarding multiple-HPV-genotype infections, and the effect of multiple-HPV infections on cervical lesions is controversial. Some studies have shown that multiple infections increase a woman's risk of cervical precancer and cancer [11–13], while others have shown that multiple HPV infections have no synergistic or additive effect on the development of cervical lesions compared to a single HPV infection [14–16].

In this study, with a method of genotyping 21 HPVs in a large quantity of cases, we investigated the prevalence and diversity of HPV infections in a routine cervical screening population in Shanghai, eastern China. We analyzed the relationship between multiple infections of various HPV genotypes and cervical lesions and aimed to further evaluate the impact of multiple-HPV infections on cervical lesion progression.

## 2. Materials and Methods

### 2.1. Study Population

With Institutional Research Review Board approval, the data for 73,596 outpatients who underwent 21-genotype HPV testing from January 2018 to April 2019 were retrieved from the database of the Department of Pathology, Obstetrics and Gynecology Hospital of Fudan University (OGHFU), including 64,534 with Pap cotest results, and 17,394 had a histological diagnosis within 6 months after HPV testing.

### 2.2. Laboratory Methods

All HPV testing was performed in the Department of Pathology at OGHFU, according to the manufacturer's instructions. HPV testing was performed by BMRT real-time PCR assay (Jiangsu BioPerfectus Technology, Taizhou, China), which genotypes 13 high-risk HPVs (hrHPVs) (HPV16, 18, 31, 33, 45, 58, 35, 52, 56, 39, 51, 68, and 59), 5 potential hrHPVs (HPV53, 66, 26, 73, and 82), and 3 low-risk HPVs (HPV6, 11, and 81) separately, and the titers were estimated by comparing to the amount of human *TOP3A* DNA. The BMRT real-time PCR assay utilizes eight tubes for one sample with three different genotypes in each tube including a reference control. In this study, only 13 types of hrHPVs and 5 types of potential hrHPVs were included in the analysis of multiple infections. Some studies have reported that the BMRT assay seemed to be a good alternative approach for HR-HPV testing, due to its high level of automation and ability to quantify HPV-16, HPV-18, and other HR-HPVs [17]. Another study has showed that BMRT is as sensitive as Cobas4800 for primary cervical cancer screening. BMRT HR-HPV viral load combined with subtypes can be used as a secondary strategy for cervical cancer screening, especially for areas with insufficient cytological resources [18].

Specimens for LBC (liquid based cytology) were obtained by a plastic spatula with an endocervical brush and were placed in transport medium. LBC was prepared via the SurePath method (BD Diagnostics) and was interpreted using the 2001 Bethesda classification system: no intraepithelial lesion or malignancy (NILM), atypical squamous cells-undetermined significance (ASC-US), atypical glandular cells (AGC), low-grade squamous intraepithelial lesions (LSIL), high-grade squamous intraepithelial lesions (HSIL), atypical squamous cells-cannot exclude HSIL (ASC-H), adenocarcinoma in situ (AIS), squamous cell carcinoma (SCC), and adenocarcinoma (ADC). Laboratory workload standards, quality control practices, and cytology-histology correlation reviews were all performed in accordance with current CAP Laboratory Accreditation Program checklists. All Pap test screenings and final interpretations were performed by pathologists in the Department of Pathology at OGHFU.

### 2.3. Statistical Analysis

Statistical analyses were performed by using IBM SPSS Statistics 20 software (IBM SPSS Institute, Inc.). The *χ*^2^ test was used to compare the ratios among the groups. The Kruskal–Wallis test and post hoc pairwise test were used to compare vial loads among groups. Logistic regressions were performed to calculate the odds ratio (OR) and 95% confident interval (CI) to assess the association of HPV subtypes and age categories with CIN2+ histopathological results (including CIN2/3, SCC, AIS, and ADC). The results were visualized by the R program using the ggplot2 package (R Core Team). Two-tailed *P* < 0.05 was considered statistically significant.

## 3. Results

### 3.1. Population Characteristics

In total, 73,596 outpatient cases were included. The age of these women ranged from 13 to 94 (mean age: 39.0 ± 11.6) years old (yo). A total of 64,534 women had Pap cotest results, of which 9.9% had low-grade cytological abnormalities (LSIL or ASC-US), and 1.3% had high-grade cytology (ASC-H, HSIL, AIS, or carcinoma). A total of 17,394 women had a histological diagnosis within 6 months after HPV testing, of whom 7.7% (1,347/17,394) had CIN2 or worse (CIN2+) lesions, and 92.3% (16,047/17,394) had less than CIN2 lesions (not CIN2+), including 17.9% (3,120/17,394) with CIN1.

### 3.2. Overall Infection Rate

**Figure 1:**
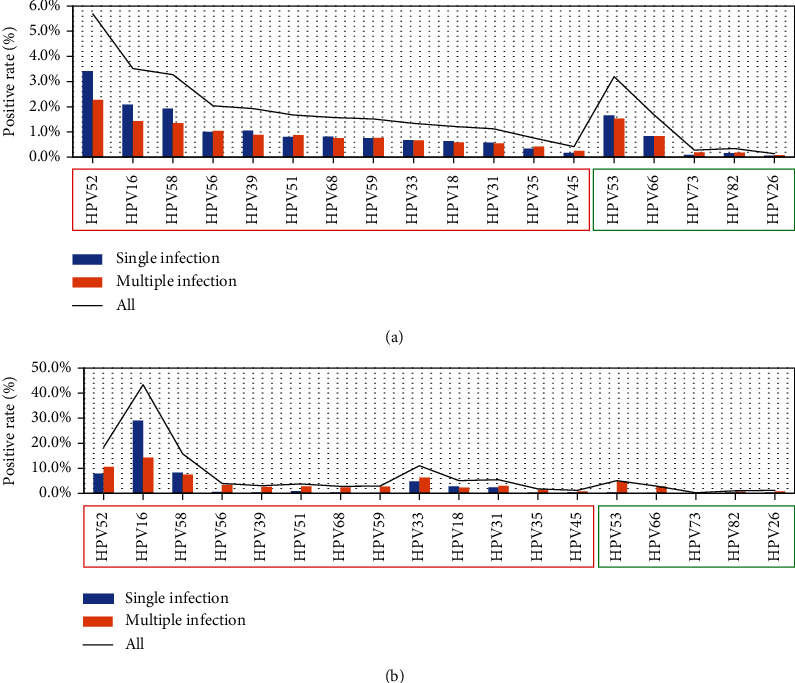
The prevalence of 13 hrHPV and 5 potential hrHPV genotypes in all patients (a) and in CIN2+ patients (b). The red box represents hrHPV genotypes, and the green box are potential hrHPV genotypes. Each genotype included single infection, multiple infections, and overall infection.

**Figure 2:**
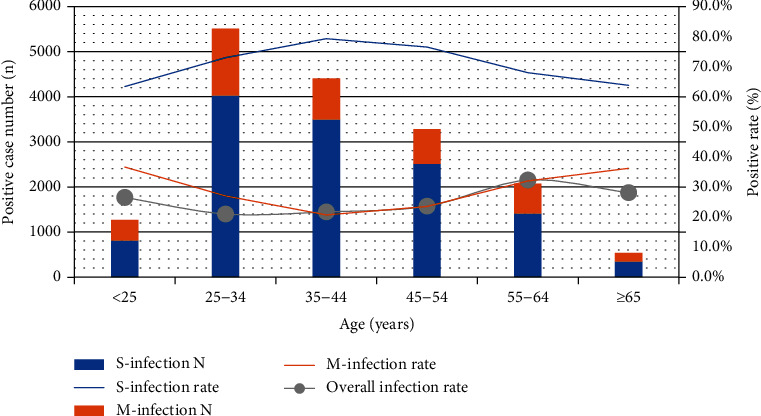
The positive case number (*N*) and positive rate of hr/phrHPV infection in different age groups. The single- (S-) infection rate and multiple- (M-) infection rate refer to the proportion of single infection and multiple infection in hr/phrHPV+ cases, respectively.

The overall infection rate of hrHPVs was 20.3% (14,913/73,596). When potential hrHPVs were added, the overall infection rate was 23.2% (17,079/73,596), and 26.3% (4,495/17,079) of which had multiple infections with as many as 9 types of hr/phrHPV. The prevalence of each genotype in descending order was HPV52>16>58>53>56>39>51>66>68>59>33>18>31>35>45>82>73>26 (Figure 1(a)). In hr/phrHPV-positive cases, the descending order of the proportion of multiple-infection was HPV73>45>35>26>82>51>56>59>66>33>31>53>68>18>39>58>16>52. HPV52 was least likely to be included in multiple infections, while HPV73 was the most. HPV73, 45, 35, 26, 82, 51, 56, and 59 were more likely to be included in multiple infections than in single infection, while other genotypes were the contrary (Figure 1(a)). The age of the women with hr/phrHPV infections ranged from 13 to 90 (mean age: 40 ± 12.343) years old (yo). In women aged <25, 25 to 34, 35 to 44, 45 to 54, 55 to 64 and ≥65 yo, the prevalence of hr/phrHPV infection was 26.5%, 21.0%, 21.7%, 23.6%, 32.2%, and 28.1%, respectively (*χ*^2^ = 449.390, *P* < 0.001), and the multiple-infection rate of hr/phrHPV was 36.6%, 27.0%, 20.7%, 23.5%, 32.1%, and 37.0%, respectively (*χ*^2^ = 217.499, *P* < 0.001) (Figure 2). Women aged <25 yo and ≥65 yo are most prone to multiple infections.

### 3.3. Prevalence of hr/phrHPV and Single- and Multiple-Infection Rates in CIN2+ Patients

In patients with CIN2+, the prevalence of hrHPV and hr/phrHPV was 87.3% (1142/1308) and 88.9% (1163/1308), respectively. The prevalence of each genotype in CIN2+ patients in descending order was HPV16>52>58>33>31>18=53>56>51>39>59=66>68>35>26=45>82>73, and among which, the single-infection rate in descending order was HPV16>58>52>33>18>31>51>56>39=26=45>53=68=82>35>59=66>73 (Figure 1(b)). HPV16, 52, 58, 33, 31, and 18 were the top six genotypes in CIN2+ patients. HPV53, a potential hrHPV, had the same prevalence as HPV18 in CIN2+ patients. And as a single infection, the prevalence of HPV 53 in CIN2+ was 0.4%, which was the same with HPV68 and higher than HPV35 and 59.

In hr/phrHPV-positive cases with CIN2+, the descending order of the proportion of multiple-infection was HPV73>53>66=59>68>39>56>35>51>82>26=45>52>33>31>58>18>16. Furthermore, only HPV16, 18, and 58 were more likely to be included in single-infection than multiple-infection in patients with CIN2+, while all other types were the contrary. And HPV73, 53, and 66(=59) were the top three genotypes most likely to be included in multiple infections (Figure 1(b)).

Single infection of HPV16 was more likely to develop into CIN2+ than multiple pattern (*P* < 0.001), indicating that mixing with other genotypes alleviated its pathogenicity. HPV52, 53, 56, 51, 39, 66, 59, 68, and 35 showed the opposite pattern (*P* < 0.001), indicating that they were less likely to be pathogens individually. All other types showed no significant differences, indicating the capability of causing diseases independently (Table 1). There was no single infection with HPV73 in CIN2+ patients, and only four patients had multiple infections with HPV73.

### 3.4. Odds Ratio (OR) for CIN2+

**Figure 3:**
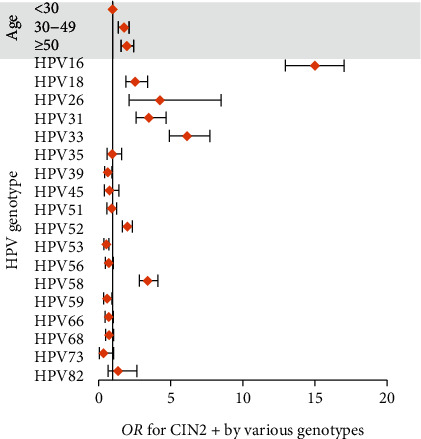
The OR for CIN2+ by age and various genotypes.

The OR of each hr/phrHPV genotype for CIN2+ is shown in Figure 3. The OR for CIN2+ increased with age. The rank of OR value for CIN2+ of each genotype was HPV16>33>26>31>58>18>52>82>35>51>45>66>56>68>59>39>53>73 (Figure 3). HPV16, 33, and 26 had the highest ORs for CIN2+, while genotypes 68, 53, and 73 had the lowest ORs. HPV26, a potential hrHPV, which has a low prevalence, showed a higher OR for CIN2+ than the majority of traditional hrHPVs. At older ages, the OR for CIN2+ was higher.

### 3.5. Viral Load of hr/phrHPVs

**Figure 4:**
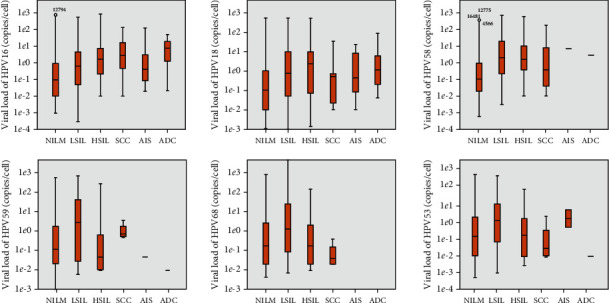
Viral load of some hr/phrHPV genotypes in different cervical lesions.

The viral loads of various types of HPV and their corresponding lesions are shown in Figure 4. The viral load of all HPV genotypes was higher in LSIL than in NILM, while comparing the viral loads between CIN2+ and NILM/LSIL, only HPV16 load increased with lesion severity (Figure 4). The viral loads of HPV53, 58, 59, and 68 in HSIL were lower than those in LSIL. The viral loads of all other types were not significantly different between LSIL and HSIL.

### 3.6. Proportion of HPV16

The correlation between single- and multiple-infection patterns of HPV16 and the pathological finding of CIN2+ lesions is shown in Table 2. As the proportion of HPV16 viral load increased, the positive rate of CIN2+ increased. This result indicates that the HPV16 load proportion was positively correlated with CIN2+ lesion development. Single infection with HPV16 was more likely to result in CIN2+ lesions than multiple infections.

## 4. Discussion

That China is vast in territory and huge in population, coupled with unbalanced development, and insufficient awareness of prevention and screening in earlier years and nonstandard cervical cancer screening in certain underdeveloped areas resulted in the regional differences in the epidemiology of HPV and cervical cancer. The overall infection rate of hrHPV in different regions of China was 8.8%-25.7% in recent years [4–6, 19–25]. Our study showed that the overall infection rate of hrHPV was 20.3%, which was similar to most reviews or researches on the nationwide prevalence of hrHPV infection in China [4–6]. In this study, HPV52, 16, and 58 were the three most common genotypes of HPV infection, consistent with the genotype distribution of hrHPV infection in China [4, 6]. HPV52, rather than HPV16, is the most popular type of HPV infection in most regions of China, especially in the East, Central, South, and Southwest China [4]. A study on the nationwide prevalence and genotype distribution of hrHPV infection in China in 2015 showed the most prevalent genotype was HPV16, followed by HPV52 [5], while a similar study in 2021 showed the reverse result [4]. The bivalent vaccine targeted toward HPV16 and HPV18 was launched and approved for use in individuals aged 9 to 45 years by the China Food and Drug Administration (CFDA) in 2016, which might be one of the possible reasons for this prevalence transition.

The prevalence of hr/phrHPV in CIN2+ patients was 88.9%, similar with the literatures [6, 25–27]. HPV16, 52, 58, 33, 31, and 18 (53) were the top six genotypes in CIN2+ patients, consistent with a meta-analysis of the prevalence and distribution of human papillomavirus genotypes in cervical intraepithelial neoplasia in China [26]. HPV53, a potential hrHPV, had the same prevalence (5.1%) as HPV18 in CIN2+ patients. The result was similar with a meta-analysis from China [26]. Furthermore, the prevalence of HPV53 ranked fourth in this study, higher than most of the hrHPV genotypes. That was consistent with the results of many studies from China [4, 21–23]. HPV53 has a high prevalence rate both in CIN2+ patients and overall population. However, the existing HPV vaccines do not target HPV53. Therefore, this study may provide a reference for future application of HPV vaccines in China.

A multicenter research from New Mexico showed that genotypes 16, 33, and 31 had the highest positive predictive values (PPVs) for CIN2+, followed by 18, 35, and 58 [28]. In the current study, we used the odds ratio (OR) and 95% confidence interval (CI) to assess the association of HPV subtypes and age categories with CIN2+ histopathological results. In this study, genotypes 16, 33, 26, 31, 58, and 18 were the top six types with the highest OR for CIN2+ (Figure 3). HPV26, a potential hrHPV, had a low prevalence but showed a higher OR for CIN2+ than the majority of traditional hrHPV. One study from our hospital showed that HPV26 was one of the top 5 CIN2+-associated HPV infection types in patients with LSIL cytology [29]. Thus, HPV26 should probably be considered a “high-risk” type. However, there are few reports related to HPV26, and the cases of HPV26 infection in this study were relatively few (65 cases). So, the result about HPV26 has certain limitations, and more cases need to be accumulated to confirm it. The prevalence of HPV73 infection in CIN2+ patients ranked last, and HPV73 had the lowest OR for CIN2+ in our data. Furthermore, there was no single infection with HPV73 in CIN2+ patients, and only four patients had multiple infections with HPV73. Therefore, HPV73 should probably be omitted from “potential hrHPV.”

To date, 20–50% of HPV-positive women have been reported to be infected with multiple HPV types [7, 10, 14, 15, 28, 30–32]. Our results showed that the multiple-infection rate in HPV-positive women was 26.3%, similar to the studies from China [4, 32]. HPV infections were significantly age-specific [6, 21]. In this study, the prevalence of hr/phrHPV infection reached a maximum in the age range from 55 to 64 yo, followed by the ≥65 group and the <25 group, while the multiple-infection rate was highest in the ≥65 group and the <25 group, followed by the 55 to 64 group (Figure 2). The hrHPV infection and multiple infections revealed the similar tendency with two peaks of infection, <25 years and 55-64 years groups in this study, conform to the epidemiology characteristics of HPV in China [4, 20–22, 33], while some studies from other countries showed that multiple-hrHPV infections were more frequent in women under 30 years of age [9, 10, 14]. The mechanisms behind the age-related infection patterns still need further study.

A study showed that clade A9 (HPV types 16, 31, 33, 35, 52, and 58) was significantly less likely to be involved in multiple infections than all other clades [31]. Another study showed that types 52, 53, 81, and 83 were more likely to occur in multiple infections with other types and that types 16, 58, and 66 were less likely to occur in multiple infections with other types [8]. In this study, HPV52, 16, 58, 39, 18, 68, 53, 31, 33, and 66 were less likely to be included in multiple infections than in single infection, while HPV73, 45, 35, 26, 82, 51, 56, and 59 were the contrary (Figure 1(a)). In CIN2+ patients, almost all genotypes were more likely to be included in multiple infections except HPV16, 18, and 58 (Figure 1(b)). On the other words, only HPV16, 18, and 58 were more likely to be enrolled in single infection in patients with CIN2+, whereas other genotypes showed reversal pattern.

In agreement with the previous research, we found that patients with single infection of HPV16 had higher incidences of CIN2+ than those with multiple-infection pattern [33], indicating that mixing with other genotypes may alleviate its pathogenicity. This finding also coincided with a study showing that multiple-HPV infections are common with no additive or synergistic effect on the development of high-risk cervical lesions [14]. Conversely, high-risk cervical lesion rates were reduced. The mechanism might be intergenotypic competition or a more effective immune response triggered by multiple infections. In this study, the infection of HPV52, 53, 56, 51, 39, 66, 59, 68, and 35 showed that multiple-infection pattern was more likely to develop into CIN2+ than single infection, indicating that these genotypes were less likely to be pathogens individually. Perhaps they coinfect with other more pathogenic genotypes. This finding was consistent with the previous study [34] and may explain studies reporting that infections with multiple HPV types seem to act synergistically in cervical carcinogenesis [35]. The infection of HPV58, 33, 31, 18, 45, 26, 82, and 73 showed no significant differences in the risk of CIN2+ between single and multiple infections in this study, indicating the capability of pathogenesis independently. This finding of us was also consistent with the previous study [15] and may explain studies reporting that the detection of multiple-HPV infections with hrHPV types is not significantly better as a predictor of cervical cancer than detection of single-hrHPV infection [36].

Studies have shown that HPV16 is the most frequently detected type and often presents with a higher viral load in cervical squamous cell carcinoma, suggesting that it could be responsible for the pathogenesis of the lesions in the majority of cases [37]. In the current study, the viral load of all HPV genotypes was higher in LSIL than in NILM; while comparing the viral load between CIN2+ and NILM/LSIL, only HPV16 load increased with lesion severity. As the proportion of HPV16 viral load increased, the positive rate of CIN2+ increased, indicating that the HPV16 load proportion was positively correlated with CIN2+ lesion development. That HPV16 single infection was more likely to result in CIN2+ lesions than multiple infections or any other genotype infection also proved that HPV16 had the strongest pathogenicity in cervical lesions.

In conclusion, extensive genotyping identified the 3 most frequent genotypes, HPV16, 58, and 52, in CIN2+ of Chinese population. Women aged younger than 25 years and older than 65 years were most prone to multiple infections. Among the hr/phrHPV-positive CIN2+ cases, HPV73, 53, and 66 (=59) were the top three genotypes most likely to be included in multiple infections, and HPV16, 18, and 58 were the 3 least. HPV26, a potential hrHPV genotype, showed a higher OR for CIN2+ lesions than most traditional hrHPV genotypes. When HPV16 mixed with other genotypes, its pathogenicity was alleviated. The vial load and the percentage of HPV16 showed a positive correlation with the severity of squamous lesions, supporting the value of extensive genotyping and viral titer quantitation. However, there are still some limitations in this study. Most of the patients in our study were from East China, and a few were from Northeast, Central, and South China. So, it cannot represent the overall prevalence of China. In addition, the number of cases of some genotype infection in CIN2+ is small, resulting in certain limitations of some results. More cases need to be accumulated for research in the future.

## Figures and Tables

**Table 1:** Comparing single- (S-) and multiple- (M-) infection patterns of hr/phrHPV genotypes.

Genotypes	Infection	CIN2+ (*N*/%)		Not CIN2+ (*N*/%)		*χ* ^2^	*P*
HPV16+	S	391	40.2%	582	59.8%	11.447	0.001
	M	193	31.7%	415	68.3%		
HPV52+	S	106	8.3%	1171	91.7%	25.810	<0.001
	M	142	15.2%	792	84.8%		
HPV53+	S	5	1.0%	507	99.0%	39.422	<0.001
	M	64	9.7%	598	90.3%		
HPV56+	S	8	2.0%	386	98.0%	25.310	<0.001
	M	46	10.7%	383	89.3%		
HPV51+	S	13	4.4%	285	95.6%	8.170	0.005
	M	38	10.3%	332	89.7%		
HPV39+	S	6	1.8%	331	98.2%	21.196	<0.001
	M	36	10.1%	319	89.9%		
HPV66+	S	3	1.1%	275	98.9%	26.296	<0.001
	M	37	11.6%	282	88.4%		
HPV59+	S	3	1.1%	267	98.9%	26.228	<0.001
	M	37	11.9%	274	88.1%		
HPV68+	S	5	1.6%	302	98.4%	21.503	<0.001
	M	33	10.6%	279	89.4%		
HPV35+	S	4	2.9%	134	97.1%	7.479	0.006
	M	21	11.0%	181	89.0%		
HPV58+	S	112	14.3%	670	85.7%	1.892	0.176
	M	101	17.0%	492	83.0%		
HPV33+	S	64	23.6%	207	76.4%	1.899	0.182
	M	85	28.7%	211	71.3%		
HPV31+	S	33	16.3%	169	83.7%	0.334	0.609
	M	41	18.5%	181	81.5%		
HPV18+	S	38	14.0%	234	86.0%	0.447	0.521
	M	31	12.0%	227	88.0%		
HPV45+	S	6	11.5%	46	88.5%	0.041	1.000
	M	11	10.5%	94	89.5%		
HPV26+	S	6	26.1%	17	73.9%	0.000	1.000
	M	11	26.2%	31	73.8%		
HPV82+	S	5	10.9%	41	89.1%	0.288	0.779
	M	10	14.3%	65	85.7%		
HPV73+	S	0	0.0%	28	100.0%	2.025	0.299
	M	4	6.9%	54	93.1%		

Not CIN2+, NILM, or LSIL.

**Table 2:** The proportion of HPV16 viral load correlated with the pathological findings of CIN2+.

The proportion of HPV16 viral load	Pathological diagnosis (*N*/%)		*χ* ^2^	*P*
	Not CIN2	CIN2+		
hrHPV(+), HPV16(-)	6458 (91.5)	599 (8.5)	924.5	<0.001
≤10%	121 (76.6)	37 (23.4)		
≤50%	94 (68.6)	43 (31.4)		
<100%	154 (63.6)	88 (36.4)		
Single infection (100%)	607 (59.7)	409 (40.3)		

## Data Availability

The data (S1 dataset) used to support the findings of this study are available in the “Supplementary Materials”.

## Supplementary Materials

Supplementary Materials S1 Dataset: the detailed data of HPVs infection.
