# Supplementary material for: Extensive HPV Genotyping Reveals High Association between Multiple Infections and Cervical Lesions in Chinese Women
Source: Dis Markers. 2022 Jun 10;2022:8130373. doi: 10.1155/2022/8130373 (PMC9205720; doi:10.1155/2022/8130373)
Supplement: Supplementary Materials — S1 Dataset: the detailed data of HPVs infection. [file 8130373.f1.docx]

Supplementary Materials:

Since the supplementary data file cannot be uploaded under the supplementary section, it has been submitted under the figures and tables section.
